# Prognostic value of right ventricular free wall strain in patients with sepsis

**DOI:** 10.3389/fcvm.2024.1334759

**Published:** 2024-02-21

**Authors:** Hongmin Chen, Lei Huang, Boyuan Xing, Yang Gao, Jie Zhang, Bingyi Zhang

**Affiliations:** ^1^ Department of Ultrasound, The First College of Clinical Medical Science, China Three Gorges University and Yichang Central People’s Hospital, Yichang, China; ^2^ Department of Critical Care Medicine, The First College of Clinical Medical Science, China Three Gorges University and Yichang Central People’s Hospital, Yichang, China

**Keywords:** strain, right ventricular dysfunction, sepsis, echocardiography, prognosis

## Abstract

**Background:**

Right ventricular systolic dysfunction (RVSD) in patients with sepsis is an area of growing interest, but its prognostic significance remains unclear and additional tools are needed to improve our understanding. Right ventricular free wall strain (RV-FWS) is a relatively new parameter to assess RV function. This study aimed to investigate the potential correlation between impaired RV-FWS and prognostic outcomes in patients with sepsis.

**Methods:**

We prospectively assessed right ventricular function in patients with sepsis within the initial 24 h of their hospital admission. RV-FWS, right ventricular global strain (RV-GS), fractional area change (FAC), and tricuspid annular plane systolic excursion (TAPSE) were examined. RVSD was defined as impaired RV-FWS. Moreover, the association between RVSD and 30-day mortality rate was assessed.

**Results:**

This study included 89 patients. Among them, 27 (30.3%) succumbed to their illness within 30 days. The nonsurviving patients demonstrated significantly lower absolute RV-FWS (−19.7% ± 2.4% vs. −21.1% ± 2.1%, *P* = 0.008) and RV-GS (−17.7% ± 1.2% vs. −18.4% ± 1.4%, *P* = 0.032) values than the surviving patients. However, TAPSE and FAC values were not significantly different between the two groups. The optimal cutoff values for RV-FWS, RV-GS, FAC, and TAPSE were −19.0%, −17.9%, 36.5%, and 1.55 cm, respectively. Kaplan–Meier survival curves revealed that patients with impaired RV-FWS and RV-GS demonstrated lower 30-day survival rates, and the predictive performance of RV-FWS (hazard ratio [HR]: 3.97, 95% confidence interval [CI]: 1.85–8.51, *P* < 0.001) was slightly higher than FAC and TAPSE. However, multivariable Cox regression analysis revealed no association between impaired RV-FWS and mortality outcomes (HR: 1.85, 95% CI: 0.56–6.14, *P* = 0.316).

**Conclusions:**

Impaired RV-FWS is not associated with short-term mortality outcomes, and RV strain imaging is of limited value in assessing the prognosis of sepsis.

## 1 Introduction

Left ventricular (LV) dysfunction is a prevalent manifestation of sepsis and is significantly correlated with an adverse prognosis (1–3). However, the function of the right ventricle (RV) has been relatively understudied. Several factors such as inflammatory cytokine activation, hypoxia/hypercapnia-induced vasoconstriction, pressure/volume overload, and myocardial ischemia can lead to RV systolic dysfunction (RVSD) in patients with sepsis (4–6). Precise assessment of RVSD is important for preventing severe right heart failure (7, 8).

At present, an internationally accepted definition of RVSD remains elusive (9, 10). Conventional echocardiography is commonly used to assess RV function; however, it may underestimate the extent of myocardial injury (11, 12). Strain imaging can accurately quantify global and segmental myocardial function by assessing the displacement of myocardial tissue. Cardiac magnetic resonance (CMR) is unaffected by acoustic windows and has a high signal-to-noise ratio, establishing it as the standard for non-invasive assessment of ventricular function (13). Within this field, techniques such as strain-encoded MR and feature tracking imaging can measure RV strain and play a role in the diagnostic classification and risk stratification of patients with heart failure (14–16). However, CMR is incompatible with metal objects such as pacemakers and infusion pumps, and patients with septic shock may have difficulties with transport and respiratory coordination.

RV-FWS, as measured by echocardiography, has been proposed to serve as a dependable indicator of RV function (17, 18); it is relatively easy to perform and has high temporal resolution. In addition, compared with traditional parameters, RV-FWS is less affected by imaging angles and demonstrates reduced reliance on LV contraction (19). However, the prognostic predictive value of RV-FWS remains inconclusive. A study conducted by Orde et al. (20) revealed that 72% of patients with sepsis demonstrated a reduction in RV-FWS, which was associated with an increased mortality rate. Conversely, Lanspa et al. (21) proposed that RV-FWS detected RVSD, but its prognostic value is limited in the context of sepsis. The debate remains ongoing. Our study aimed to explore the performance of RV function parameters (RV-FWS, RV-GS, TAPSE, and FAC) in predicting mortality outcomes in patients with sepsis and investigate the association between RVSD defined by RV-FWS and 30-day mortality.

## 2 Materials and methods

### 2.1 Study population

We prospectively enrolled adult patients with sepsis who had received treatment at the Yichang Central People's Hospital from May 2019 to April 2023. Sepsis is a life-threatening organ dysfunction caused by a dysregulated host response to infection. Septic shock is sepsis with persisting hypotension requiring vasopressors to maintain a mean arterial pressure of ≥65 mmHg and a serum lactate level of >2 mmol/L despite adequate volume resuscitation (22). The exclusion criteria of this study were as follows: patients with severe valvular disease or previous valve surgery; those with acute coronary syndrome (<1 month); those with persistent or permanent atrial fibrillation; those with other etiologies causing RV remodeling, such as RV cardiomyopathy and pulmonary arterial hypertension; and those with suboptimal ultrasound image quality or missing data. The hospital's institutional ethics committee approved this study (Approval No: PJ-KY2020-27). Patients or their legal guardians provided informed consent.

### 2.2 Echocardiography

Transthoracic echocardiography (TTE) (GE, Vivid E9, Horten Norway) measurements were completed within the first 24 h after admission. Conventional echocardiographic parameters were assessed according to the guidelines set by the American Society of Echocardiography (ASE) (23). Tricuspid annular plane systolic excursion (TAPSE) was measured at the junction of the lateral tricuspid leaflet and RV-free wall using the M-mode. The areas of RV systolic and diastolic were assessed in the RV-focused apical 4-chamber view. Fractional area change (FAC) was calculated using the following formula: [(end-diastolic area − end-systolic area)/end-diastolic area] × 100%. Inferior vena cava (IVC) diameter and its collapse with respiration were measured in the sagittal view. The ASE guidelines considered an IVC diameter of >2.1 cm and/or a collapse rate of <50% as positive findings. The right atrial pressure was estimated as 8 mmHg with one positive indicator, 15 mmHg with two positive indicators, and 3 mmHg with no positive indicators (23).

**Figure 1:**
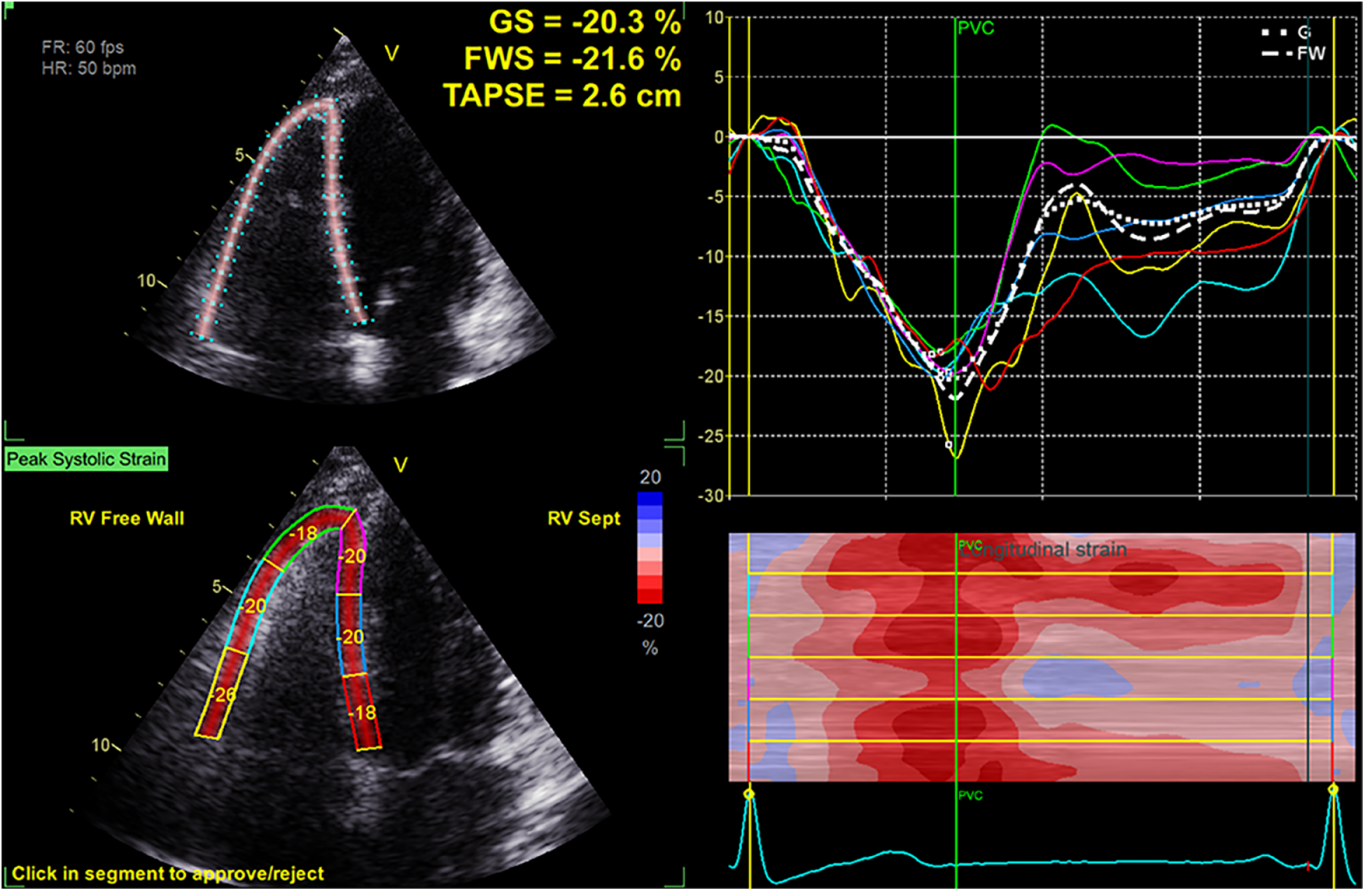
Measurement of RV-FWS and RV-GS in an RV-focused apical four-chamber view.

Dynamic images capturing a minimum of three consecutive cardiac cycles were saved (in DICOM format) in the RV-focused apical 4-chamber view. Strain analysis was conducted using the EchoPac workstation (GE, version 204, Horten Norway). A region of interest was established following the selection of the tricuspid annulus and apical plane, and the tracing curve was manually fine-tuned to achieve optimal alignment. The software algorithm automatically computed the RV-GS, RV-FWS, and strain values for each myocardial segment (Figure 1). To minimize information bias, data collection was performed by two experienced sonographers who were blinded to the clinical data.

### 2.3 Clinical data

In addition to vital demographic information, we collected data on patients' temperature; heart rate; respiratory rate; site of infection upon admission; and biochemical parameters, such as myocardial injury markers and lactate levels. We sought the expertise of the attending physician to assess the patient's renal injury and conduct a sequential organ failure assessment (SOFA). Furthermore, we documented the mean arterial pressure and mechanical ventilation utilization during TTE. Norepinephrine was the primary vasopressor medication. Our primary clinical outcome was 30-day mortality.

### 2.4 Statistical analysis

Continuous variables following a normal distribution are expressed as mean ± standard deviation, whereas those with a non-normal distribution are represented as median (interquartile range). Categorical variables are presented as percentages. Independent sample *t*-test, Mann–Whitney *U*-test, chi-square (*χ*^2^) test, or Fisher's exact test was used to analyze disparities between the groups. Receiver operating characteristic (ROC) curve analysis was performed for parameters related to RV function. Cutoff values were determined based on the maximum value of Youden's index, and the data were subsequently converted into binary variables. The constructed Kaplan–Meier survival curves and Log-Rank test were used to analyze the disparities among various groups. Univariate and multivariate Cox regression models were used to assess the association between variables and 30-day mortality. The same and different operators randomly analyzed 10 patients; the analyses were spaced at least 1 week apart to assess the intraoperator and interoperator reproducibility of strain measurements. Repeatability was quantified using the intraclass correlation coefficient (ICC). A *P*-value of <0.05 was considered statistically significant. The Statistical Package for the Social Sciences software version 26 (IBM Corp., Armonk, NY, USA) was used for all statistical analyses.

## 3 Results

### 3.1 General characteristics

**Figure 2:**
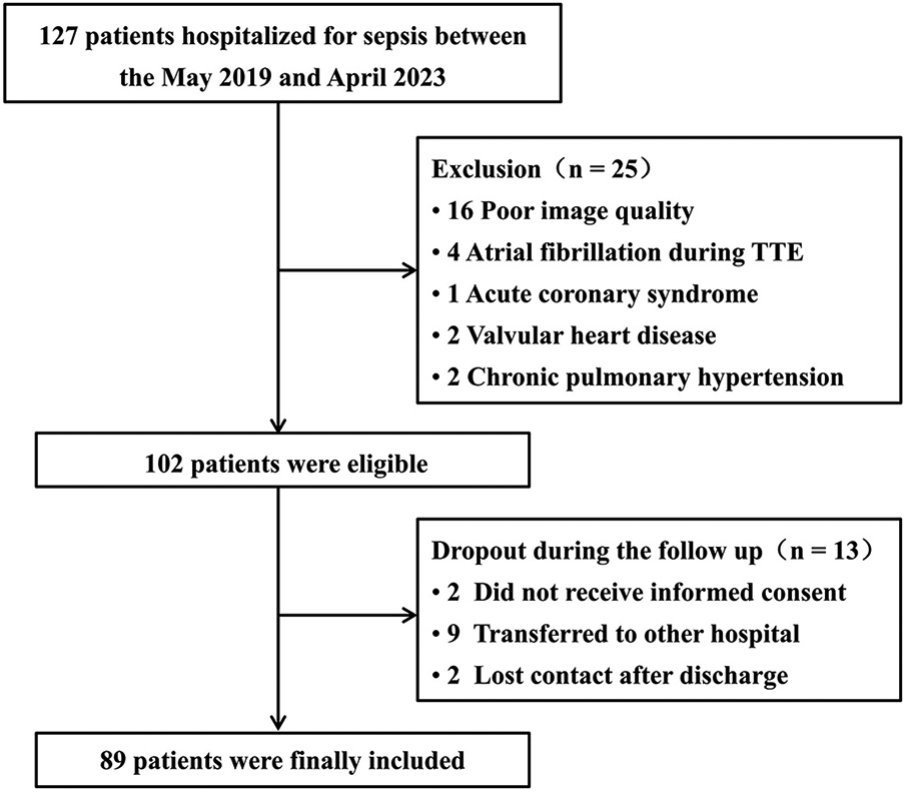
Flow diagram showing the initial selection of the cohort and exclusion of patients. TTE, transthoracic echocardiography.

In total, 127 patients were hospitalized due to sepsis conditions. Of these, 38 (30%) were excluded, predominantly due to substandard ultrasound image quality (*n* = 16) (Figure 2). Finally, 89 patients (median age: 63.0 ± 11.6 years) were enrolled in the study (Figure 2), including 59.6% males. Of the enrolled patients, 54 (61%) were diagnosed with septic shock and required vasopressor support, 27 (30%) patients died within 30 days of admission (Table 1). Mechanical ventilation was administered to 44 (49%) patients. Acute renal insufficiency was observed in 17 (19%) patients, and renal replacement therapy was initiated in 5 (6%) patients. One (1%) patient developed infective endocarditis. Furthermore, while 9 patients were transferred to other hospitals (Figure 2), subsequent surveys revealed a 30-day mortality rate of approximately 33% for these patients, which is consistent with our findings, mitigating selection bias to some extent.

**Table 1:** Clinical and TTE parameters according to survival outcomes.

Variable	All patients (*n* = 89)	Alive (*n* = 62)	Dead (*n* = 27)	*P-*value
Sex (male, %)	53 (59.6)	37 (59.7)	16 (59.3)	0.971
Age (year)	63.0 ± 11.6	60.2 ± 11.5	68.7 ± 9.1	0.001
Cardiovascular risk factors				
Hypertension (%)	29 (36.6)	17 (27.4)	12 (44.4)	0.115
Diabetes (%)	15 (16.9)	11 (17.7)	4 (14.8)	0.735
Coronary heart disease (%)	12 (13.5)	8 (12.9)	4 (14.8)	0.808
COPD (%)	9 (10.1)	4 (6.5)	5 (18.5)	0.083
Chronic renal disease (%)	5 (5.6)	4 (6.5)	1 (3.7)	0.605
Infection site				
Respiratory system (%)	27 (30.3)	18 (29.0)	9 (33.3)	0.685
Digestive system (%)	27 (30.3)	16 (25.8)	11 (40.7)	0.159
Urinary system (%)	15 (16.9)	13 (21.0)	2 (7.4)	0.116
Superficial tissue (%)	8 (9.0)	6 (9.7)	2 (7.4)	0.731
Other (%)	12 (13.5)	9 (14.5)	3 (11.1)	0.665
Body mass index (kg/m^2^)	23.8 ± 3.6	23.5 ± 3.5	24.6 ± 3.7	0.261
Heart rate (bpm)	108.0 ± 12.0	104.5 ± 12.5	110.8 ± 9.8	0.024
Respiratory rate (bpm)	21.0 ± 3.1	21.3 ± 2.7	22.3 ± 3.8	0.172
Temperature (°C)	37.5 (37.0, 38.2)	37.6 (37.2, 38.0)	37.5 (36.8, 38.3)	0.897
Mean arterial pressure (mmHg)	77.0 ± 11.7	78.8 ± 11.4	73.2 ± 11.8	0.038
Lactate concentration (mmol/L)	2.9 (2.0, 4.2)	2.5 (1.8, 3.9)	3.6 (2.7, 4.6)	0.024
NT-pro BNP (pg/ml)	574 (182, 921)	328 (128, 619)	1,178 (663, 2,925)	<0.001
Cardiac troponin I (µg/ml)	0.16 (0.05,0.44)	0.11 (0.04, 0.21)	1.02 (0.24, 2.64)	<0.001
Sequential organ failure assessment	7 (5, 11)	6 (4, 8)	10 (9, 12)	<0.001
Vasopressors during TTE (yes, %)	54 (60.7)	33 (53.2)	21 (77.8)	0.029
Ventilated during TTE (yes, %)	44 (49.4)	26 (41.9)	18 (66.7)	0.032
Acute kidney injury (yes, %)	17 (19.1)	8 (12.9)	9 (33.3)	0.024
Right atrium pressure (mmHg)	3 (3, 8)	3 (3, 8)	8 (3, 8)	0.016
RV-free wall strain (%)	−20.4 ± 2.3	−21.1 ± 2.1	−19.7 ± 2.4	0.008
RV globe strain (%)	−18.2 ± 1.4	−18.4 ± 1.4	−17.7 ± 1.2	0.032
Fractional area change (%)	36.3 ± 3.0	36.6 ± 2.9	35.4 ± 3.2	0.092
TAPSE (cm)	1.70 ± 0.27	1.79 ± 0.27	1.67 ± 0.25	0.052
LV ejection fraction (%)	56 (49, 58)	57 (50, 60)	55 (48, 57)	0.041
LV end-diastolic volume (ml)	111.0 ± 17.8	106.8 ± 16.8	112.9 ± 19.5	0.137
LV end-systolic volume (ml)	49.0 ± 12.7	48.8 ± 11.4	54.7 ± 14.7	0.041
E (cm/s)	81.9 ± 18.3	80.6 ± 17.2	82.9 ± 20.9	0.594
e' (cm/s)	6.4 ± 1.4	6.7 ± 1.4	6.4 ± 1.6	0.282
E/e'	12.4 ± 2.3	12.3 ± 2.4	13.3 ± 2.2	0.060

COPD, chronic obstructive pulmonary disease; NT-pro BNP, N-terminal pro-B-type natriuretic peptide; TTE, transthoracic echocardiography; RV, right ventricular; TAPSE, tricuspid annular plane systolic excursion; LV, left ventricular.

Table 1 outlines patient characteristics based on survival outcomes. Nonsurvivors were significantly older and presented higher SOFA scores and increased markers of myocardial damage as well as a greater incidence of acute kidney injury (AKI) than survivors. Vasopressor medication administration (77.8% vs. 53.2%, *P* = 0.029) and mechanical ventilation (66.7% vs. 41.9%, *P* = 0.032) were more prevalent among nonsurvivors than survivors. Furthermore, nonsurvivors demonstrated lower absolute values of RV-FWS (−21.1% ± 2.1% vs. −19.7% ± 2.4%, *P* = 0.008) and RV-GS (−18.4% ± 1.4% vs. −17.7% ± 1.2%, *P* = 0.032) than survivors. TAPSE (1.79 ± 0.27 cm vs. 1.67 ± 0.25 cm, *P* = 0.052) and FAC (36.6% ± 2.9% vs. 35.4% ± 3.2%, *P* = 0.092) demonstrated no significant differences between the two groups (Table 1).

### 3.2 Prognostic value of different ultrasonographic parameters

**Figure 3:**
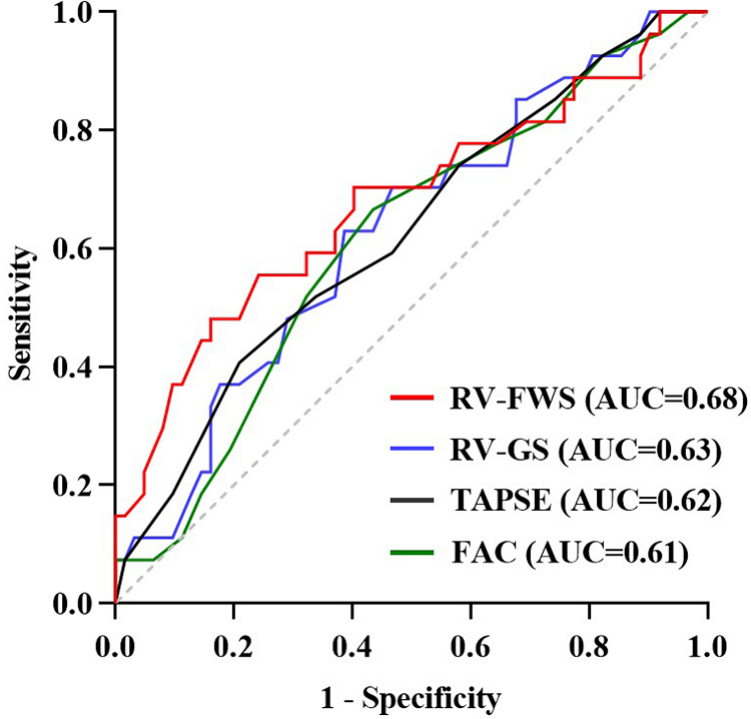
ROC curve analysis of RV function parameters for predicting 30-day mortality.

**Figure 4:**
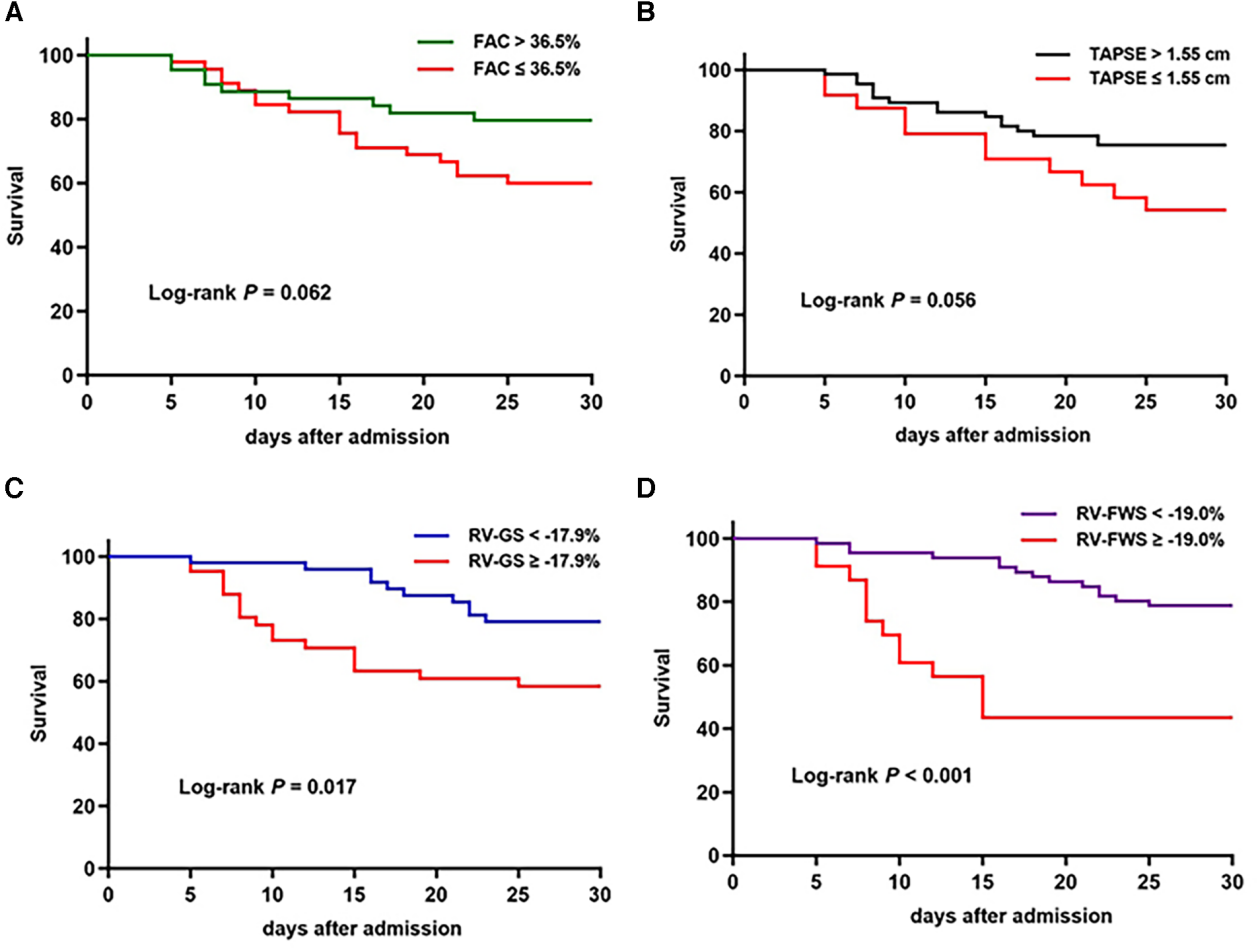
Kaplan–Meier survival curves based on FAC (**A**), TAPSE (**B**), RV-GS (**C**), and RV-FWS (**D**) cutoff values.

The ROC analysis included RV-FWS, RV-GS, FAC, and TAPSE and revealed optimal cutoff values of −19.0%, −17.9%, 36.5%, and 1.55 cm, respectively. Of these factors, RV-FWS demonstrated the largest area under the curve (AUC: 0.68, *P* = 0.008) (Figure 3). The Kaplan–Meier survival analysis revealed that patients with impaired RV-FWS and RV-GS demonstrated a reduced survival rate compared with those with preserved RV-FWS and RV-GS. Neither FAC (*P *= 0.062) nor TAPSE (*P* = 0.056) significantly differentiated patients with different outcomes (Figure 4). However, in the multivariate analyses, neither of the two strain parameters, RV-FWS and RV-GS, was found to be associated with increased mortality (Table 2).

**Table 2:** Univariate and multivariate Cox analyses of variables associated with 30-day mortality.

Variable	Univariate HR (95% confidence interval)	*P*-value	Multivariate HR (95% confidence interval)	*P*-value
Sex (male, %)	1.02 (0.47–2.20)	0.960		
Age (year)	1.06 (1.02–1.10)	0.002	0.98 (0.92–.04)	0.462
Body mass index (kg/m^2^)	1.07 (0.96–1.19)	0.225		
Heart rate (bpm)	1.03 (0.99–1.06)	0.061		
Respiratory rate (bpm)	1.08 (0.96–1.21)	0.195		
Temperature (°C)	0.92 (0.56–1.50)	0.730		
Mean arterial pressure (mmHg)	0.96 (0.92–1.01)	0.037	0.97 (0.92–1.03)	0.355
Vasopressors during TTE (yes, %)	2.37 (1.07–5.29)	0.034	0.79 (0.18–3.39)	0.748
Ventilated during TTE (yes, %)	2.36 (1.06–5.26)	0.036	1.82 (0.69–4.79)	0.223
Acute kidney injury (yes, %)	2.89 (1.30–6.46)	0.009	2.25 (0.85–5.99)	0.103
Sequential organ failure assessment	1.16 (1.07–1.27)	0.001	1.15 (1.00–1.33)	0.046
Lactate concentration (mmol/L)	1.27 (1.03–1.55)	0.024	1.06 (0.69–1.63)	0.780
NT-pro BNP ≧632 (pg/ml)	12.1 (4.20–35.0)	<0.001	7.54 (2.28–22.96)	<0.001
Cardiac troponin I ≧0.215 (µg/ml)	8.08 (3.25–20.9)	<0.001	8.67 (2.57–29.29)	0.001
RV globe strain ≧−17.9%	2.49 (1.14–5.45)	0.022	1.32 (0.44–3.99)	0.621
RV-free wall strain ≧−19.0%	3.97 (1.85–8.51)	<0.001	1.85 (0.56–6.14)	0.316
Right atrium pressure (mmHg)	1.13 (1.03–1.24)	0.008	0.98 (0.84–1.15)	0.822
LV ejection fraction (%)	1.02 (0.99–1.04)	0.146		
LV ejection fraction ≦50%	1.25 (0.55–2.85)	0.603		
LV end-diastolic volume (ml)	1.03 (1.00–1.06)	0.053		
LV end-systolic volume (ml)	0.96 (0.91–1.01)	0.128		
E (cm/s)	1.01 (0.98–1.03)	0.533		
e' (cm/s)	0.87 (0.66–1.15)	0.346		
E/e'	1.15 (0.99–1.33)	0.066		

NT-proBNP, N-terminal pro-B-type natriuretic peptide; TTE, transthoracic echocardiography; RV, right ventricular; LV, left ventricular.

### 3.3 RVSD and mortality outcome

RVSD was defined by RV-FWS of ≥−19.0%. In the present study, 23 (25.8%) patients with RVSD had a higher mortality rate (56.5% vs. 21.2%, *P* = 0.002). Furthermore, individuals with RVSD demonstrated an increased prevalence of AKI (34.8% vs. 13.6%, *P* = 0.026) and mechanical ventilation (73.9% vs. 40.9%, *P* = 0.006), elevated lactate levels and SOFA scores, as well as a reduced left ventricular ejection fraction (LVEF) (Table 3). However, RVSD did not appear as an independent mortality predictor after adjusting for variables such as age, mechanical ventilation, AKI, myocardial damage markers, and SOFA score in the Cox regression analysis [hazard ratio (HR): 1.85, 95% confidence interval (CI): 0.56–6.14, *P* = 0.316] (Table 2). In addition, 13 (14.6%) patients demonstrated isolated RVSD (LVEF >50%), and this subgroup of patients demonstrated a significant association with the 30-day mortality outcome (HR: 3.46, 95% CI: 1.22–9.82, *P* = 0.020). The incidence rate of biventricular dysfunction was approximately 11% (*n* = 10). However, no significant relationship was observed between biventricular dysfunction and mortality outcome (Table 4).

**Table 3:** Clinical and TTE parameters according to the presence or absence of RVSD.

Variable	No RVSD (*n* = 66)	RVSD (*n* = 23)	*P*-value
30-day mortality (%)	14 (21.2)	13 (56.5)	0.002
Sex (male, %)	36 (54.5)	17 (73.9)	0.103
Age (year)	62 (54, 67)	68 (61, 76)	0.018
Cardiovascular risk factors			
Hypertension (%)	19 (28.8)	10 (43.5)	0.196
Diabetes (%)	11 (16.7)	4 (17.4)	0.936
Coronary heart disease (%)	10 (15.2)	2 (8.7)	0.670
Chronic obstructive pulmonary disease (%)	5 (7.6)	4 (17.4)	0.179
Chronic renal disease (%)	4 (6.1)	1 (4.3)	0.615
Infection site			
Respiratory system (%)	17 (25.8)	10 (43.5)	0.111
Digestive system (%)	23 (34.8)	4 (17.4)	0.117
Urinary system (%)	10 (15.2)	5 (21.7)	0.687
Superficial tissue (%)	7 (10.6)	1 (4.3)	0.631
Other (%)	9 (13.6)	3 (13.0)	0.626
Body mass index (kg/m^2^)	23.8 ± 3.8	24.2 ± 2.9	0.660
Heart rate (bpm)	106.1 ± 13.1	107.3 ± 8.6	0.612
Respiratory rate (bpm)	21 (20, 23)	22 (20, 24)	0.193
Temperature (°C)	37.6 ± 0.8	37.6 ± 0.9	0.965
Mean arterial pressure (mmHg)	78 (71, 87)	69 (63, 79)	0.005
Lactate concentration (mmol/L)	2.6 (1.8, 3.9)	3.5 (2.8, 5.1)	0.009
NT-pro BNP (pg/ml)	461 (150, 750)	690 (258, 1,935)	0.021
Cardiac troponin I (µg/ml)	0.13 (0.04, 0.25)	0.29 (0.13, 2.18)	0.023
Sequential organ failure assessment	7 (4, 10)	9 (7,13)	0.014
Vasopressors during TTE (yes, %)	39 (59.1)	15 (65.2)	0.604
Ventilated during TTE (yes, %)	27 (40.9)	17 (73.9)	0.006
Acute kidney injury (yes, %)	9 (13.6)	8 (34.8)	0.026
Right atrium pressure (mmHg)	3 (3, 8)	8 (6, 8)	<0.001
Left ventricular ejection fraction (%)	56 (50, 60)	52 (47, 56)	0.004
Left ventricular ejection fraction ≦50% (%)	19 (28.7)	10 (43.4)	0.196
Left ventricular end-diastolic volume (ml)	108.0 ± 17.3	110.5 ± 19.3	0.567
Left ventricular end-systolic volume (ml)	49.1 ± 12.2	54.9 ± 13.2	0.055
E (cm/s)	82.9 ± 18.5	76.5 ± 17.5	0.151
e' (cm/s)	6.7 ± 1.4	6.3 ± 1.5	0.156
E/e'	12.6 ± 2.4	12.8 ± 2.3	0.726

NT-pro BNP, N-terminal pro-B-type natriuretic peptide; TTE, transthoracic echocardiography.

**Table 4:** Univariate and multivariate Cox analyses of variables associated with 30-day mortality according to LVEF subgroups.

Variable	Univariate HR (95% confidence interval)	*P*-value	Multivariate HR (95% confidence interval)	*P*-value
Left ventricular ejection fraction >50% (*n* = 60)				
Cardiac troponin I ≧0.215 (µg/mL)	5.97 (1.97–18.06)	0.002	5.16 (1.64–16.27)	0.005
NT-pro BNP ≧632 (pg/mL)	17.47 (4.01–76.15)	<0.001	11.97 (2.65–54.07)	0.001
Sequential organ failure assessment	1.16 (1.04–1.29)	0.008		
Right ventricular free wall strain ≧−19.0% (*n* = 13)	7.62 (2.99–19.44)	<0.001	3.46 (1.22–9.82)	0.020
Left ventricular ejection fraction ≦50% (*n* = 29)				
Cardiac troponin I ≧0.215 (µg/mL)	20.51 (3.89–107.99)	<0.001	25.54 (3.59–181.34)	0.001
NT-pro BNP ≧632 (pg/mL)	6.08 (1.22–30.27)	0.028		
Sequential organ failure assessment	1.19 (1.04–1.37)	0.011	1.29 (1.04–1.61)	0.023
Right ventricular free wall strain ≧−19.0% (*n* = 10)	1.30 (0.31–5.45)	0.720		

NT-proBNP, N-terminal pro-B-type natriuretic peptide.

RV-FWLS demonstrated high intraobserver and interobserver reproducibility, with ICC values of 0.89 (95% CI: 0.72–0.96) and 0.85 (95% CI: 0.61–0.94), respectively (Table 5).

**Table 5:** Reproducibility of RV-FWS and RV-GS.

	ICC for intraobservers	95% CI	ICC for interobservers	95% CI
RV-FWS (%)	0.89	0.72–0.96	0.85	0.61–0.94
RV-GS (%)	0.91	0.78–0.96	0.84	0.60–0.94

RV-FWS, right ventricle free wall strain; RV-GS, right ventricle globe strain.

## 4 Discussion

This observational study on sepsis revealed the following results: (1) RVSD defined by RV-FWS was present in 26% of the study patients, and their mortality rate was over double that of patients without RVSD; (2) nonsurvivors had significantly lower absolute values of RV-FWS than the survivors, and RV-FWS may provide added value in identifying RVSD; (3) in multivariate analyses, RV-FWS did not demonstrate an independent association with increased mortality; (4) isolated RVSD (with normal LVEF) may be correlated with prognosis. Further research with a larger sample size is warranted to validate these findings.

RVSD is frequently observed in patients with sepsis (20, 21, 24, 25). Vallabhajosyula et al. applied TAPSE and FAC as indices to quantify RVSD, revealing a 55% prevalence in patients with sepsis (26), a figure apparently exceeding the 26% incidence observed in our cohort (Table 3). This variation could be associated with different RVSD definitions and TTE evaluation timing. We defined RVSD using an RV-FWS threshold of −19%, which may have potentially biased the cohort toward individuals with pronounced RV impairment and possibly neglected subjects with incipient or progressive dysfunction. Recent research has revealed that RV-FWS may serve as a reliable indicator of RV systolic function (27, 28). Although RV-free wall motion occurs primarily in the transverse direction (29), strain analysis enables tracking of myocardial displacement from multiple orientations and is less affected by imaging angle limitations (30). Furthermore, the interventricular septum provides an anatomical basis for the interdependence of biventricular functional contraction (31). Compared with RV-GS, RV-FWS shows less dependence on LV motion (17, 32). These features highlight the advantage of RV-FWS in evaluating myocardial function.

Infection causes reversible or irreversible damage to the microcirculation and endothelial cells, resulting in the release of inflammatory mediators and myocardial depressant factors into the bloodstream. This can induce partial myocardial cell apoptosis and a negative inotropic effect to a certain extent (5, 33). Significantly elevated myocardial injury biomarkers in patients with sepsis support this hypothesis (Table 3). However, the pathophysiological mechanisms responsible for RVSD are complex, and cardiomyocyte injury alone does not entirely account for this phenomenon. The RV's long-term low-pressure ejection makes it less tolerant to acute increases in afterload (34, 35). We revealed an increased lactate level and relatively insufficient tissue perfusion in patients with RVSD (Table 3). Hypoxia and acidosis-induced pulmonary vasoconstriction may cause increased RV afterload (36, 37). Furthermore, while we did not observe a correlation between mechanical ventilation and increased mortality, the use of mechanical ventilation also leads to an increase in pulmonary vascular resistance to some extent (38, 39). In addition, individuals with RVSD demonstrated elevated right atrial pressure, emphasizing the effect of volume overload. Therefore, further research is required to determine whether RVSD in sepsis is a concomitant phenomenon of increased load.

The prognostic significance of RVSD remains unclear. Orde et al. (20) revealed that nonsurvivors demonstrated significantly impaired RV-FWS compared with survivors. RV-FWS of >−13% persisted as an independent predictor of 6-month mortality in patients with sepsis after adjusting for factors such as mechanical ventilation. Our study revealed that the mortality rate for patients with RV-FWS of ≥−19% increased by 2.7 times (*P* = 0.002) (Table 3), but impaired RV-FWS was not a determinant of mortality outcomes after adjusting for clinically pertinent covariates (Table 2). The use of an RV-focused apical 4-chamber view for strain analysis may have caused our higher absolute values of strain. Moreover, our research incorporated more covariate parameters, and we used a short follow-up period, which may have mitigated the effect of secondary diseases and treatment modalities on survival outcomes. Lanspa et al. (21) adopted RV-FWS of −20% as the threshold and revealed no correlation between impaired RV-FWS and 28-day mortality in patients with sepsis. Our study corroborates their findings and also demonstrates that the presence of isolated RVSD (with preserved LVEF) may increase the risk of mortality (Table 4). Isolated RVSD has been suggested as a maladaptive response to stress that may be associated with the development of secondary cardiogenic shock in sepsis (40, 41). We used LVEF to assess LV systolic function and found that the incidence of LV systolic dysfunction in patients with sepsis was approximately 32% (Table 4). However, in multivariate analysis, neither LV systolic dysfunction nor biventricular systolic dysfunction emerged as a risk factor for mortality outcomes (Tables 2, 4). The prognostic value of LVEF in sepsis is limited and it may not be a reliable indicator of intrinsic myocardial contractile function (42, 43), particularly in the context of interventions, such as aggressive fluid resuscitation or vasoactive medications. In addition, some studies have suggested that concurrent biventricular dysfunction is a tolerable adaptive response (44, 45).

Our study had several limitations. First, we did not include assessment data for the RV outflow tract, thereby the effect of RV-pulmonary artery decoupling was not investigated. Second, we could not evaluate the trend of changes in RV function as treatment progressed due to the lack of follow-up examinations during hospitalization. Third, although we used consistent diagnostic criteria, the Berkson bias remained. In addition, we did not eliminate the potential impact of confounding factors such as secondary lung infection, antibiotic use, and fluid balance on patient outcomes. Fourth, LV performance was assessed by LVEF rather than strain. Fifth, this single-center study included a relatively small sample size and we did not perform a power analysis. Furthermore, strain measurement necessitates high-quality images (17), and different analysis software demonstrated substantial heterogeneity (46).

## 5 Conclusion

In the present study, 26% of patients with sepsis had RVSD, which was defined as RV-FWS of ≥−19%. Nonsurvivors exhibited a higher incidence of RVSD than survivors. However, impaired RV-FWS was not identified as an independent risk factor for 30-day mortality, suggesting that RVSD may not affect the short-term prognosis of patients with sepsis. To validate this finding, research with larger sample sizes and multicentre designs is required in the future.

## Data Availability

The original contributions presented in the study are included in the article/Supplementary Material, further inquiries can be directed to the corresponding author.
